# Clinically Relevant Doses of Remimazolam Modulate Cardiac Electrophysiology: Late Repolarization Prolongation and Increased Conduction Dispersion With Preserved QTc

**DOI:** 10.1155/crp/9479974

**Published:** 2026-04-13

**Authors:** Zijun Wang, Ying Cao, Gao Su, Yanyan Feng, Rongfeng Yang, Xue Bai, Hong Gao

**Affiliations:** ^1^ School of Anesthesia, Guizhou Medical University, Guiyang, 550004, Guizhou, China, gmc.edu.cn; ^2^ Department of Anesthesiology, The Second People’s Hospital of Guiyang, Guiyang, 550081, Guizhou, China, jyhosp.cn; ^3^ Translational Medicine Research Center, Guizhou Medical University, Guiyang, 561113, Guizhou, China, gmc.edu.cn; ^4^ Department of Anesthesiology, The Affiliated Hospital of Guizhou Medical University, Guiyang, 550004, Guizhou, China, gmcah.cn

**Keywords:** action potential, calcium transient, cardiac electrophysiology, conduction, remimazolam

## Abstract

**Background:**

Remimazolam, an ultra‐short‐acting benzodiazepine with rapid metabolism and cardiovascular stability, is increasingly used for anesthesia, yet its cardiac electrophysiologic effects are incompletely characterized.

**Methods:**

We conducted a multimodal evaluation in Langendorff‐perfused guinea pig hearts and human induced pluripotent stem cell‐derived cardiomyocytes (hiPSC‐CMs), using surface electrocardiogram (ECG), multielectrode mapping, optical mapping, and whole‐cell patch‐clamp across remimazolam doses (0, 1, 2, 3 mg/kg/h).

**Results:**

High‐dose remimazolam (3 mg/kg/h) prolonged the PR interval (*p* = 0.027), and T‐wave duration was prolonged at 2 and 3 mg/kg/h (*p* = 0.017 and *p* < 0.001), while QT interval and corrected QT interval (QTc) remained unchanged (*p* > 0.1). Multielectrode mapping showed prolonged activation time at 2 and 3 mg/kg/h versus NC (*p* = 0.02 and 0.003) and increased conduction dispersion at 2 and 3 mg/kg/h (*p* = 0.0193 and 0.0101). Conduction velocity (CV) was reduced at 3 mg/kg/h compared with NC and 1 mg/kg/h (*p* = 0.01 and 0.02). Optical mapping demonstrated prolonged action potential duration at 90% repolarization (APD_90_) (NC: 107.93 ± 0.63 ms vs 3 mg/kg/h: 118.94 ± 1.83 ms, *p* < 0.001) and calcium transient duration at 90% recovery (CTD_90_) (NC: 116.20 ± 1.04 ms vs 3 mg/kg/h: 125.63 ± 1.15 ms, *p* < 0.001), accompanied by increased APD_90_ interquartile range (APD_90_‐IQR; *p* ≤ 0.02 vs NC) and increased CTD_90_ interquartile range (CTD_90_‐IQR; *p* = 0.005). In hiPSC‐CMs, 1500 ng/mL remimazolam selectively prolonged APD_90_ (*p* = 0.04) without significant effects on action potential amplitude, upstroke velocity, or early repolarization indices (all *p* > 0.05).

**Conclusion:**

These findings indicate that at higher clinically relevant exposures, remimazolam selectively lengthens late repolarization and increases conduction heterogeneity—features consistent with an arrhythmogenic substrate—while QT and QTc remains stable, supporting cautious use and ECG monitoring in at‐risk populations.

## 1. Background

Anesthetic agents can potentially influence cardiac electrophysiology, occasionally triggering perioperative arrhythmias and compromising patient safety [1–3]. Benzodiazepines are commonly utilized in clinical anesthesia owing to their anxiolytic, sedative, and hypnotic properties; however, concerns remain regarding their cardiac conduction and electrophysiological stability [4, 5]. Remimazolam, a novel ultra‐short‐acting benzodiazepine, has emerged as a promising sedative agent due to its favorable pharmacological profile, including rapid onset, short duration of action, and predictable pharmacokinetics [6–8]. Following rigorous Phase III clinical trials, remimazolam was first approved for clinical use in 2020 for the induction and maintenance of general anesthesia and procedural sedation [9–11]. Its unique metabolism via rapid hydrolysis by carboxylesterase‐1 into an inactive metabolite (CNS 7054) ensures high plasma clearance, minimal accumulation, and rapid patient recovery after sedation, making it particularly advantageous for patients with hepatic or renal impairments [12, 13].

Current knowledge of remimazolam predominantly highlights its rapid metabolism, minimal accumulation, and favorable pharmacodynamics in sedation and general anesthesia. Clinical trials consistently indicate good cardiovascular stability, positioning remimazolam as beneficial for patients with cardiovascular comorbidities or in intensive care units [14–16]. A hemodynamic study comparing remimazolam to propofol demonstrated similar modest reductions in mean arterial pressure, heart rate (HR), cardiac output, and stroke volume during anesthesia induction, with no significant differences observed between these anesthetic agents [15]. Preclinical studies in animal models indicated dose‐dependent yet transient cardiovascular effects, primarily characterized by moderate reductions in mean arterial pressure and compensatory increases in HR [17].

Electrophysiological assessments, however, present a significant knowledge gap. Regulatory guidelines stress the critical importance of cardiac repolarization assessments during drug development to identify potential proarrhythmic effects [17]. Although clinical electrocardiogram (ECG) studies have shown no significant changes in intervals or waveform morphology during remimazolam administration, in vitro investigations revealed concentration‐dependent effects on human ether‐à‐go‐go‐related gene (hERG) potassium channels at substantially higher concentrations than clinical plasma levels [8, 18]. Given the scarcity of studies and the inconsistency of existing findings, a comprehensive evaluation of the electrophysiological safety profile of remimazolam is urgently needed.

Therefore, this study aims to systematically investigate the cardiac electrophysiological effects of continuous infusion of different concentrations of remimazolam. Utilizing multichannel electrical and optical mapping techniques in isolated perfused hearts, we assessed critical electrophysiological parameters, including conduction velocity (CV), directional propagation, conduction heterogeneity, action potential duration (APD), and calcium transient (CaT) signals. Additionally, whole‐cell patch‐clamp techniques were employed to measure APD in human induced pluripotent stem cell‐derived cardiomyocytes (hiPSC‐CMs), offering cellular‐level insights. These methodological approaches collectively aim to establish a comprehensive safety profile, bridging the gap between preclinical observations and clinical applicability, thus ensuring safe clinical use of remimazolam.

## 2. Materials and Methods

### 2.1. Ethical Approval

All experimental procedures were approved by the Animal Ethics Committee of Third Affiliated Hospital of Guizhou Medical University, China (No. 2021A010), and were conducted in accordance with national guidelines and the NIH Guide for the Care and Use of Laboratory Animals (8th edition).

### 2.2. Experimental Animals

Male guinea pigs (250–320 g) were purchased from the Experimental Animal Center of Guizhou Medical University. Animals were housed at 23°C with 60% humidity, under a 12/12‐h light/dark cycle, and had free access to food and water.

### 2.3. hiPSC‐CMs

hiPSC‐CMs were purchased from Beijing Saibei Biological Co. and cultured in matrix gel–coated dishes. The differentiation protocol involved initial treatment with GSK3 inhibitor CHIR99021 followed by the Wnt signaling inhibitor IWP2. hiPSC‐CMs between Days 40 and 60 of differentiation were isolated using type I collagenase and 0.25% trypsin and seeded onto 3.5‐cm culture dishes for subsequent electrophysiological studies.

### 2.4. Drug Preparation and Solutions

Remimazolam (No. 220308AU, Jiangsu Hengrui Pharmaceutical Co., Jiangsu, China) was reconstituted in sterile 0.9% saline to achieve required concentrations. Krebs–Henseleit (K–H) solution composition was as follows (mM): 119 NaCl, 25 NaHCO_3_, 4 KCl, 1 MgCl_2_, 1.2 KH_2_PO_4_, 1.8 CaCl_2_·2H_2_O, and 10 D‐glucose. Extracellular solution for hiPSC action potential recordings contained (mM) 127 NaCl, 5.9 KCl, 2.4 CaCl_2_, 1.2 MgCl_2_, 11 glucose, and 10 HEPES (pH 7.4 adjusted with NaOH). Pipette solution included (mM) 10 HEPES, 126 KCl, 6 NaCl, 1.2 MgCl_2_, 5 EGTA, 11 glucose, and 1 MgATP (pH 7.2 adjusted with KOH).

### 2.5. Langendorff‐Perfused Isolated Hearts

Guinea pigs were anesthetized with 2.5%–3.0% isoflurane inhalation until loss of pedal reflexes was confirmed. Hearts were rapidly excised, mounted, and retrogradely perfused using a Langendorff apparatus at a flow rate of 8 mL/min with K–H solution maintained at 37°C. Hearts were stabilized for 20 min prior to experiments.

### 2.6. Multichannel Electrical Mapping

Guinea pig hearts were randomly divided into four groups: normal control (NC, *n* = 8), 1 mg/kg/h (*n* = 7), 2 mg/kg/h (*n* = 6), and 3 mg/kg/h (*n* = 6). Hearts were perfused continuously for 2 hours. Two cascaded 64‐channel multielectrode array systems (EMS64‐USB‐1003, MappingLab Ltd., UK) were used to record extracellular potentials (ECPs) from the epicardial surface. An 8 × 8 microelectrode array (MappingLab Ltd., UK) was placed on the left ventricular free wall to record local field potentials. The signals were amplified and digitized at a 10 kHz sampling frequency and displayed using EMapRecord 5.7.7 software (MappingLab Ltd., UK). ECG signals were recorded continuously using two electrodes positioned on the surface of the right atrium and the apex of the left ventricle, respectively, and were amplified using a MappingLab filter amplifier. Activation time was defined as the point of maximum negative slope of the unipolar electrogram. Parameters including CV, conduction time, and conduction heterogeneity index were calculated using EMapScope 5.8.1 software (MappingLab Ltd., UK).

### 2.7. Optical Mapping Techniques

Guinea pig hearts were perfused at 37°C and 10 mL/min using a Langendorff system. Following stabilization, contractions were suppressed by 10 μmol/L blebbistatin (Abcam, ab120425). To enhance dye uptake, 20% Pluronic F‐127 (Invitrogen, P3000MP) was perfused prior to dye loading. For dual‐parameter optical mapping, RH237 (Chem Cruz, sc‐499456; 1 μg/mL) and Rhod‐2 AM (Abcam, ab142780; 1 μg/mL) were introduced sequentially. Illumination was provided by 530 nm light‐emitting diode (LED) (LEDC‐2001, MappingLab Ltd.) with appropriate bandpass filters (530 ± 20 nm). Emitted fluorescence was optically separated using a 638 nm dichroic mirror. Fluorescence above 638 nm passed through a 700 nm long‐pass filter for membrane potential (Vm) signals; light below 638 nm passed through a 585 ± 40 nm bandpass filter for CaT. Data were acquired with the OMS‐PCIE‐2002 EMCCD system (MappingLab Ltd.) at 128 × 128 pixel resolution, 16 × 16 mm field of view, and 900 frames/s. LED illumination, electrical stimulation, ECG, and recording were synchronized via an 8‐channel TTL controller. All signals were captured using OMapRecord 4.0 and analyzed offline with OMapScope 5.0 for isochronal mapping and quantitative analysis of Vm and CaT dynamics.

### 2.8. Whole‐Cell Patch‐Clamp Technique

hiPSC‐CMs were placed in a temperature‐controlled chamber (35°C–37°C) and continuously perfused with extracellular solution. Borosilicate pipettes (3–5 MΩ resistance) were filled with pipette solution. Action potentials were recorded using current‐clamp mode at 1 Hz stimulation (800 pA for 2 ms) with a Multiclamp 700B amplifier (Axon, USA). Parameters assessed included resting membrane potential (RMP), action potential amplitude (APA), maximum upstroke velocity (Vmax), and APD. Remimazolam concentrations corresponding to clinical pharmacokinetics were 1.14, 2.28, and 3.42 µM.

### 2.9. Statistical Analysis

All statistical analyses were performed using GraphPad Prism 10 (GraphPad Software, San Diego, CA, USA). Electrophysiological datasets were processed and visualized using Clampfit 10.6 (Molecular Devices) and OriginPro 9.0 (OriginLab). Data are presented as mean ± standard error of the mean (SEM). Normality was assessed using the Shapiro–Wilk test. For comparisons among multiple groups, one‐way analysis of variance (ANOVA) was applied for normally distributed data, followed by Tukey’s multiple‐comparisons test. For datasets that did not meet normality assumptions, the Kruskal–Wallis test was used, followed by Dunn’s multiple‐comparisons test. All tests were two‐tailed, and *p* < 0.05 was considered statistically significant. Statistical significance is indicated as ^∗^ *p* < 0.05, ^∗∗^ *p* < 0.01, and ^∗∗∗^ *p* < 0.001.

## 3. Results

### 3.1. High‐Dose Remimazolam Prolongs PR Interval and T Wave Duration in Isolated Guinea Pig Hearts

To systematically evaluate the electrophysiological effects of remimazolam, ECG parameters (HR, PR interval, QRS duration, QT interval, corrected QT interval [QTc], and T wave duration) were assessed in isolated guinea pig hearts. All experimental groups maintained sinus rhythm throughout the recording. The PR interval was significantly prolonged only at the highest dose (3 mg/kg/h) compared with NC (*p* = 0.027), increasing from 69.36 ± 1.92 to 78.13 ± 1.90 ms, corresponding to an absolute increase of 8.77 ms. T‐wave duration was significantly prolonged at both 2 mg/kg/h (*p* = 0.017) and 3 mg/kg/h (*p* < 0.001) versus NC, increasing from 50.23 ± 4.29 to 73.17 ± 4.45 ms (+22.94 ms) and 86.32 ± 7.75 ms (+36.09 ms), respectively; T wave duration was also greater at 3 mg/kg/h than at 1 mg/kg/h (60.00 ± 3.45 ms; *p* = 0.007), with an absolute difference of 26.32 ms. By contrast, HR and QRS duration did not differ significantly among groups (all *p* > 0.1). QT interval showed only a modest numerical increase across remimazolam doses (NC: 190.50 ± 4.48 ms; 1 mg/kg/h: 207.77 ± 8.61 ms; 2 mg/kg/h: 199.17 ± 6.27 ms; 3 mg/kg/h: 195.33 ± 6.71 ms), but between‐group differences were not statistically significant (*p* > 0.1). QTc likewise remained stable (NC: 142.41 ± 6.04 ms; 1 mg/kg/h: 139.92 ± 9.41 ms; 2 mg/kg/h: 131.97 ± 6.23 ms; 3 mg/kg/h: 142.76 ± 7.34 ms; *p* > 0.1). Sample sizes for each group are provided in Figure 1 legend. Representative ECG tracings demonstrated PR interval prolongation and dose‐related T wave broadening with increased amplitude, consistent with the quantitative analyses (Figure 1).

FIGURE 1Effects of different doses of remimazolam on ECG parameters in isolated guinea pig hearts. (a) Representative ECG tracings recorded under normal control (NC) conditions and during remimazolam perfusion at 1, 2, and 3 mg/kg/h, illustrating PR‐interval prolongation and T‐wave broadening at higher doses. (b–h) Quantitative comparisons of heart rate (b), PR interval (c), QRS duration (d), QT interval (e), T‐wave duration (f), QTc interval (g), and QT/QTc ratio (h) across groups. Isolated hearts were assigned to NC (*n* = 8), 1 mg/kg/h (*n* = 7), 2 mg/kg/h (*n* = 6), or 3 mg/kg/h (*n* = 6). Statistical comparisons were performed using one‐way ANOVA followed by Tukey’s post hoc test. Data are presented as mean ± SEM; ^∗^ *p* < 0.05, ^∗∗^ *p* < 0.01, and ^∗∗∗^ *p* < 0.001.

**Figure:**
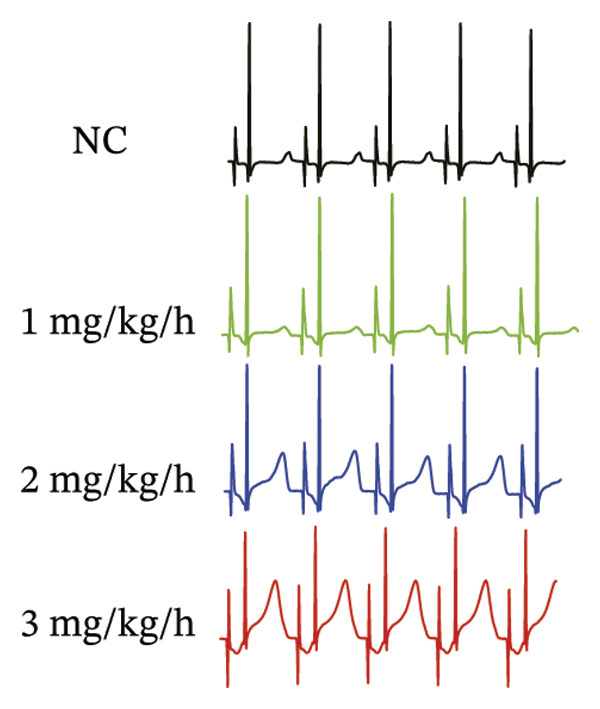
(a)

**Figure:**
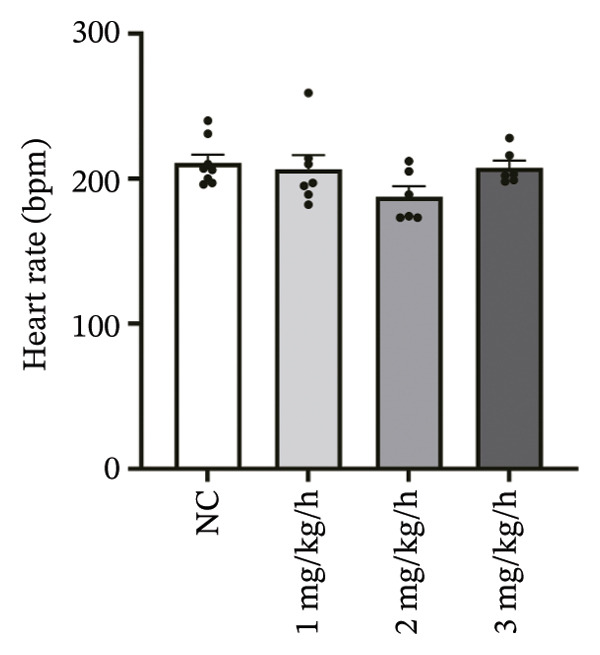
(b)

**Figure:**
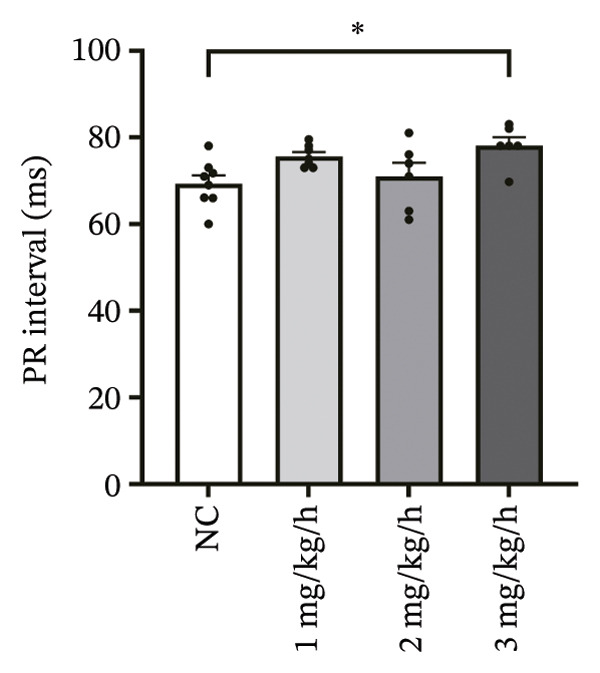
(c)

**Figure:**
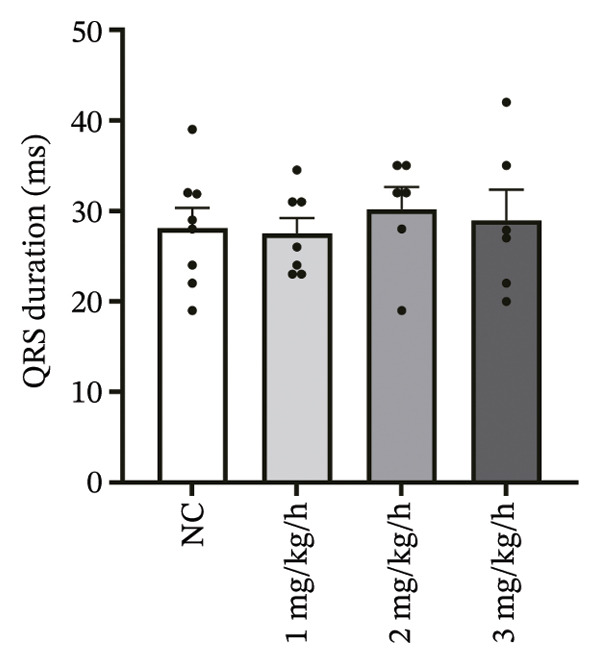
(d)

**Figure:**
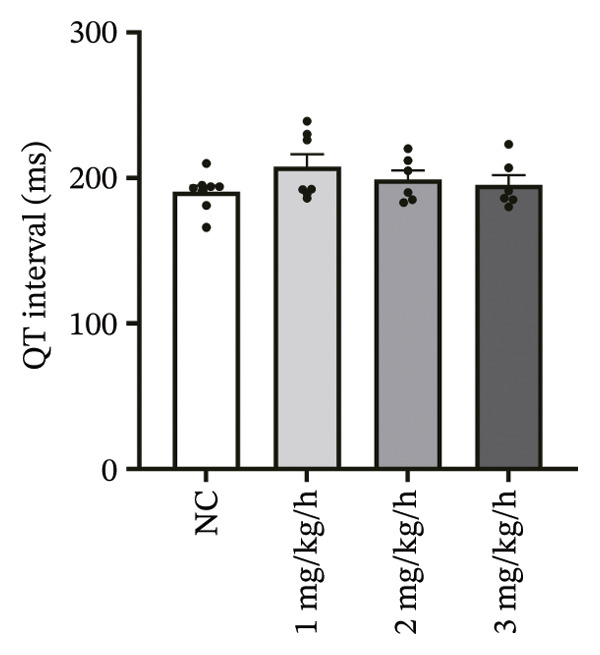
(e)

**Figure:**
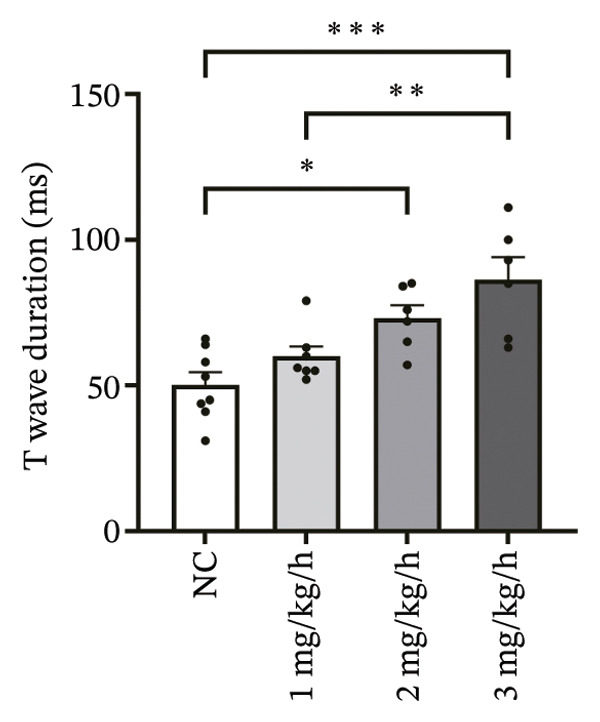
(f)

**Figure:**
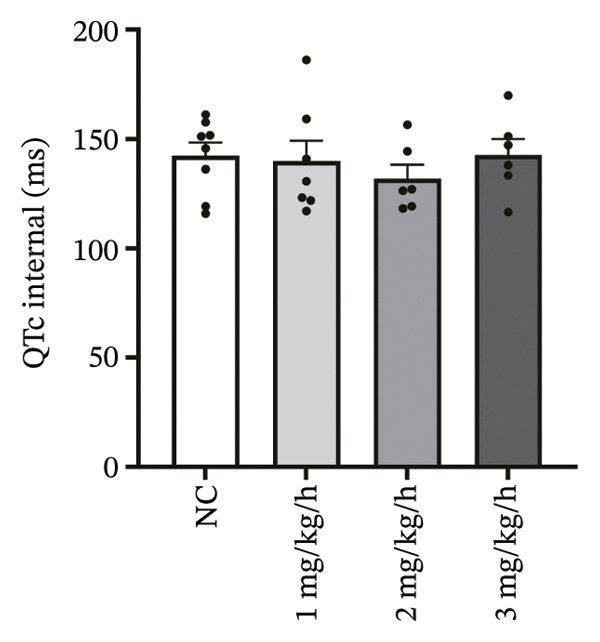
(g)

**Figure:**
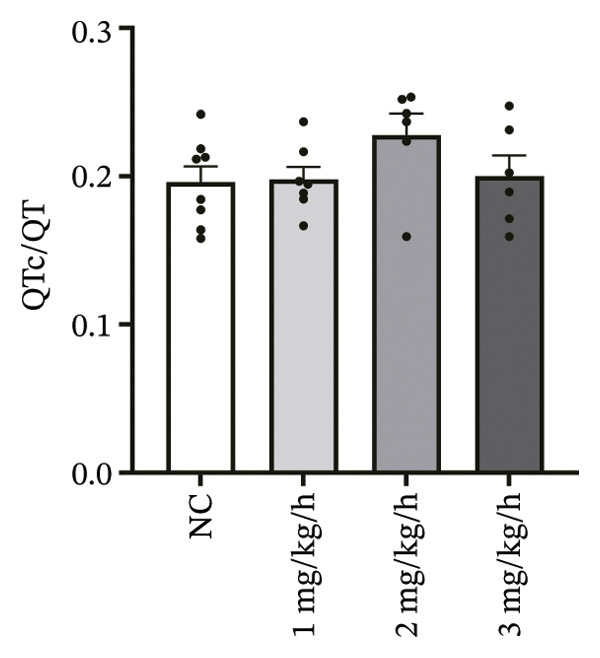
(h)

### 3.2. High‐Dose Remimazolam Increases Ventricular Conduction Heterogeneity

To investigate the impact of remimazolam on ventricular conduction uniformity, multielectrode mapping was performed in Langendorff‐perfused guinea pig hearts to quantify activation time, conduction dispersion, inhomogeneity index, absolute inhomogeneity, and CV. Activation time increased at higher doses and was significantly prolonged at 2 and 3 mg/kg/h compared with NC (*p* = 0.02 and 0.003), rising from 1.22 ± 0.19 ms (NC) to 4.43 ± 0.97 ms (2 mg/kg/h) and 5.27 ± 0.79 ms (3 mg/kg/h). Conduction dispersion was significantly higher at 2 mg/kg/h and 3 mg/kg/h versus NC (*p* = 0.0193 and 0.0101), increasing from 0.60 ± 0.09 ms to 2.59 ± 0.49 ms and 2.70 ± 0.78 ms, respectively. The inhomogeneity index did not differ significantly among groups (all *p* > 0.05), although it showed a numerical upward trend at 2 mg/kg/h (2.25 ± 0.23) and 3 mg/kg/h (3.35 ± 0.63) versus NC (2.09 ± 0.27). Absolute inhomogeneity was markedly greater at 3 mg/kg/h than NC (*p* = 0.002), increasing from 1.05 ± 0.23 mm/ms to 6.19 ± 1.17 mm/ms, whereas the difference at 2 mg/kg/h did not reach statistical significance (*p* = 0.07). CV was significantly reduced at 3 mg/kg/h compared with NC and 1 mg/kg/h (*p* = 0.01 and 0.02), decreasing from 2.12 ± 0.51 mm/ms (NC) and 2.09 ± 0.49 mm/ms (1 mg/kg/h) to 0.64 ± 0.08 mm/ms (3 mg/kg/h). Collectively, these mapping results indicate that higher clinically relevant remimazolam exposure delays activation and increases spatial conduction nonuniformity, consistent with an arrhythmia‐prone conduction substrate (Figure 2).

FIGURE 2Effects of remimazolam on ventricular conduction heterogeneity in Langendorff‐perfused guinea pig hearts. (a) Representative activation time map showing increased regional activation delays with higher remimazolam doses. (b) Representative conduction dispersion map illustrating enhanced spatial variability in activation timing. (c–g) Group comparisons of activation time (ms) (c), conduction velocity, CV (mm/ms) (d), conduction dispersion (ms) (e), inhomogeneity index (dimensionless) (f), and absolute inhomogeneity (mm/ms) (g) in isolated hearts perfused with NC (*n* = 8), 1 mg/kg/h (*n* = 7), 2 mg/kg/h (*n* = 6), or 3 mg/kg/h (*n* = 6) remimazolam. Activation time was significantly increased at 2 and 3 mg/kg/h versus NC (Tukey’s multiple comparisons). Conduction dispersion was significantly increased at 2 and 3 mg/kg/h versus NC (Dunn’s multiple comparisons), whereas the inhomogeneity index did not differ significantly among groups. Absolute inhomogeneity was significantly increased at 3 mg/kg/h versus NC (Dunn’s multiple comparisons), while the difference at 2 mg/kg/h did not reach significance. CV was significantly reduced at 3 mg/kg/h compared with NC and 1 mg/kg/h (Dunn’s multiple comparisons). Data are presented as mean ± SEM; ^∗^ *p* < 0.05; ^∗∗^ *p* < 0.01.

**Figure:**
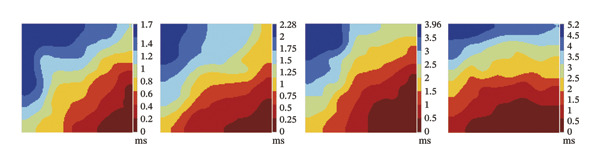
(a)

**Figure:**
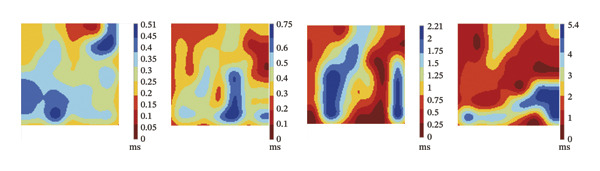
(b)

**Figure:**
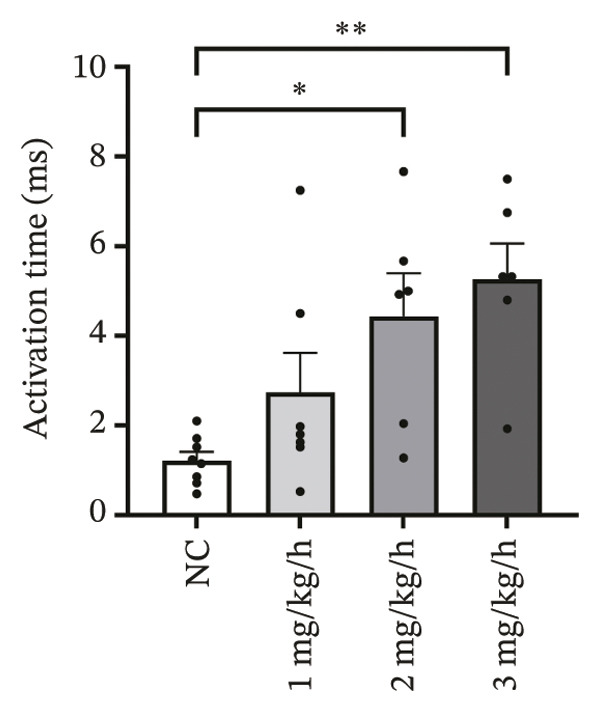
(c)

**Figure:**
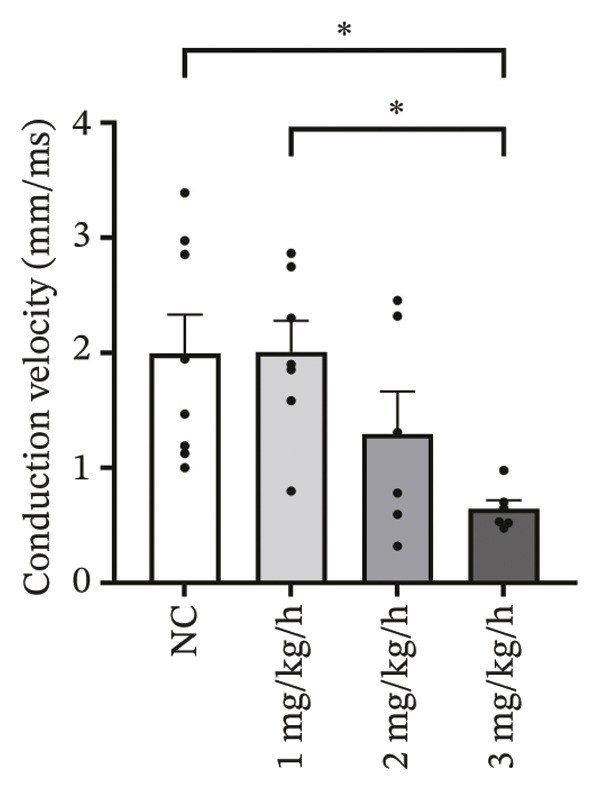
(d)

**Figure:**
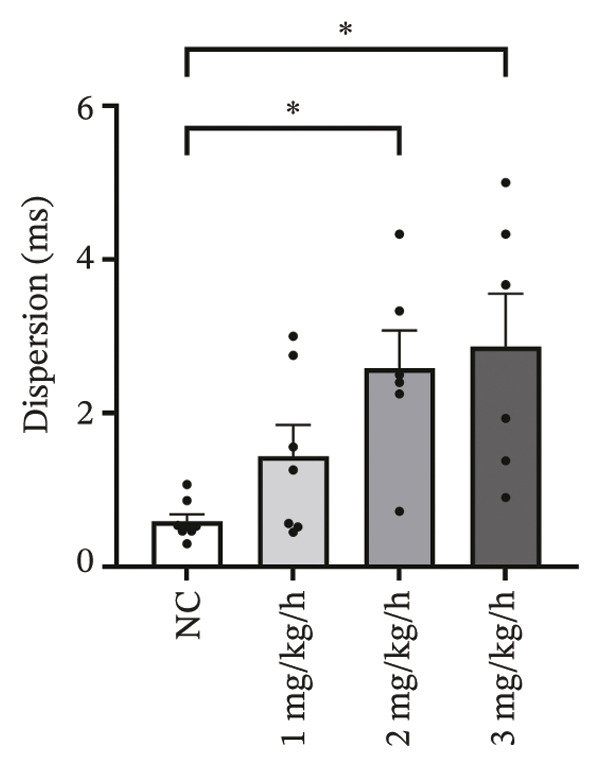
(e)

**Figure:**
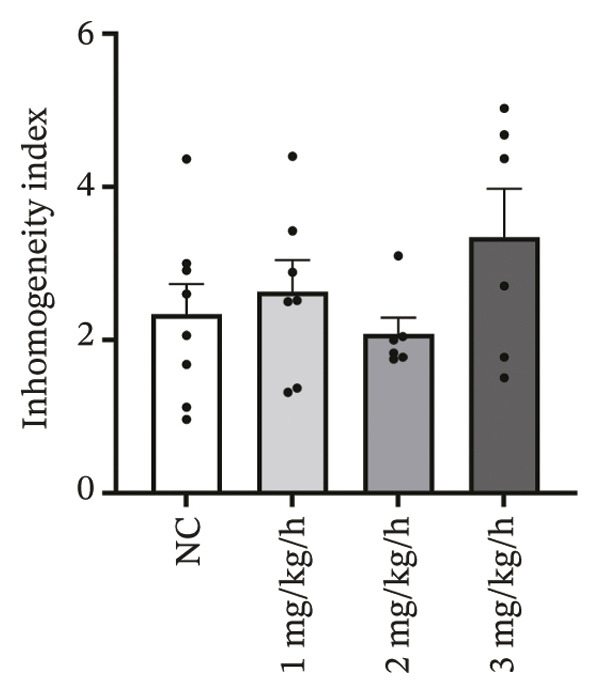
(f)

**Figure:**
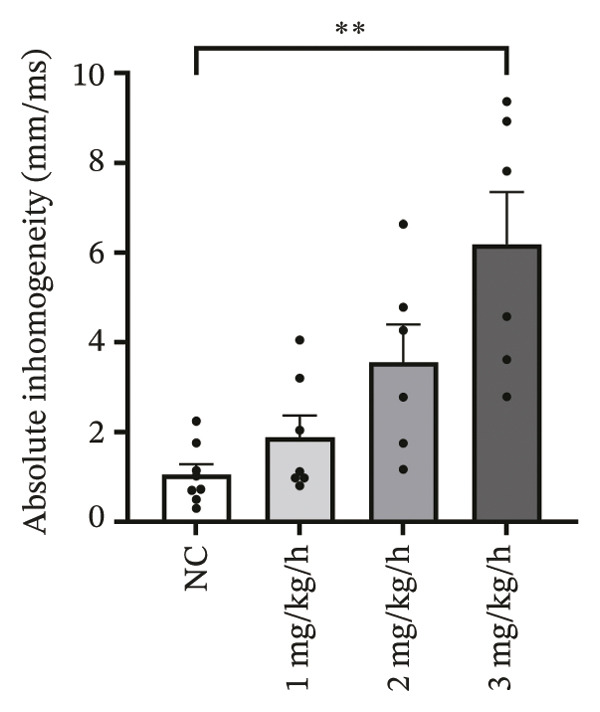
(g)

### 3.3. Remimazolam Dose‐Dependently Alters Action Potentials and CaTs

To minimize intrinsic rhythm variability, all optical mapping recordings were standardized with constant electrical pacing at 4.5 Hz in Langendorff‐perfused guinea pig hearts. Under these conditions, AP and CaT parameters were evaluated systematically. Remimazolam induced a dose‐dependent prolongation in AP rise time. Compared with NC (25.74 ± 0.58 ms), rise time was significantly increased at 1 mg/kg/h (29.81 ± 0.49 ms, *p* < 0.001), 2 mg/kg/h (30.30 ± 0.53 ms, *p* < 0.001), and 3 mg/kg/h (32.31 ± 0.85 ms, *p* < 0.001). APD_90_ was also prolonged across doses (NC: 107.93 ± 0.63 ms; 1 mg/kg/h: 115.81 ± 1.74 ms, *p* = 0.006; 2 mg/kg/h: 117.22 ± 1.72 ms, *p* = 0.001; 3 mg/kg/h: 118.94 ± 1.83 ms, *p* < 0.001). Spatial heterogeneity, quantified as APD_90_‐IQR, increased across doses and was higher than NC at 1 mg/kg/h (14.76 ± 0.83 ms vs 11.15 ± 0.47 ms, *p* = 0.02), 2 mg/kg/h (17.40 ± 0.76 ms, *p* < 0.001), and 3 mg/kg/h (19.33 ± 1.13 ms, *p* < 0.001); additionally, 3 mg/kg/h exceeded 1 mg/kg/h (*p* = 0.003), whereas differences between 1 and 2 mg/kg/h (*p* = 0.13) and between 2 and 3 mg/kg/h (*p* = 0.38) were not significant. Activation time and APD_30–80_ remained stable across groups (all *p* > 0.05) (Figure 3).

FIGURE 3Effects of remimazolam on action potential characteristics in Langendorff‐perfused guinea pig hearts. All optical mapping recordings were obtained under constant electrical pacing at 4.5 Hz to minimize variability due to intrinsic rhythm. (a–c) Representative maps of activation time (a), rise time (b), and action potential duration at 90% repolarization (APD_90_) (c) under NC conditions and during remimazolam perfusion at 1, 2, and 3 mg/kg/h. (d–h) Group comparisons of activation time (d), rise time (e), APD_90_ (f), APD_90_ interquartile range (IQR) (g), and APD_30–80_ duration (h) across groups. (i) Overlaid representative action potential traces from each group illustrating dose‐dependent kinetic changes. *n* = 9 hearts per group. Statistical comparisons were performed using one‐way ANOVA followed by Tukey’s post hoc test. Data are presented as mean ± SEM; ^∗^ *p* < 0.05, ^∗∗^ *p* < 0.01, and ^∗∗∗^ *p* < 0.001.

**Figure:**
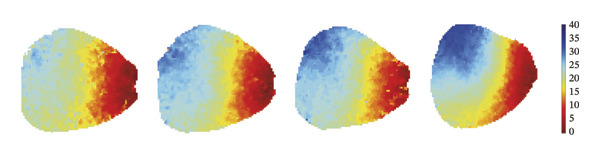
(a)

**Figure:**
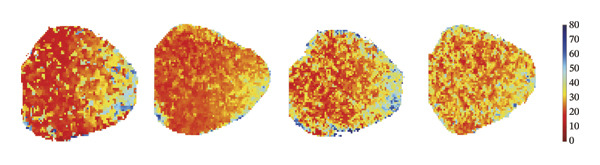
(b)

**Figure:**
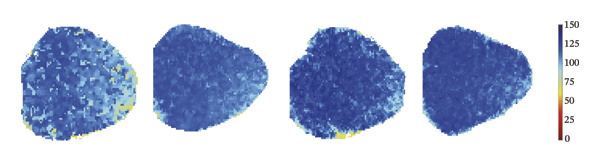
(c)

**Figure:**
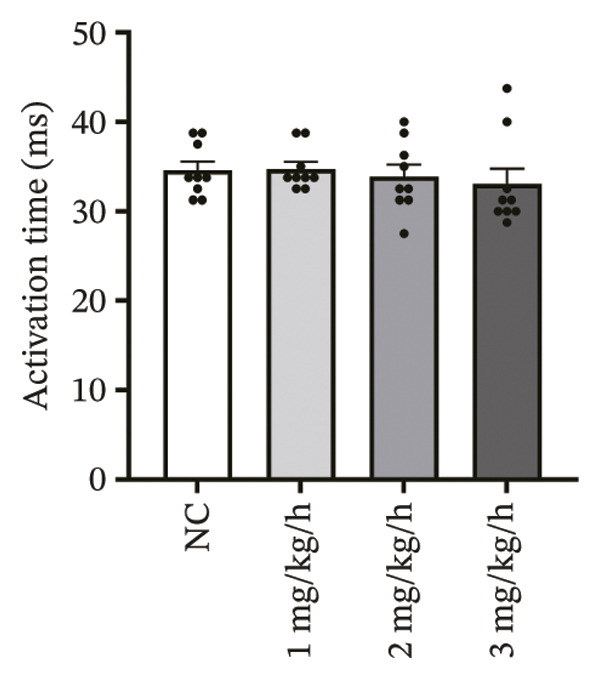
(d)

**Figure:**
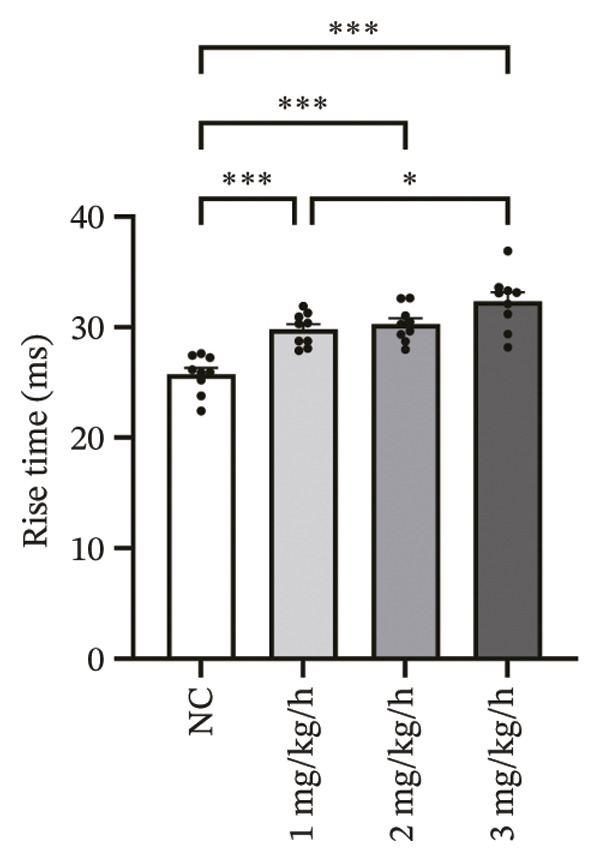
(e)

**Figure:**
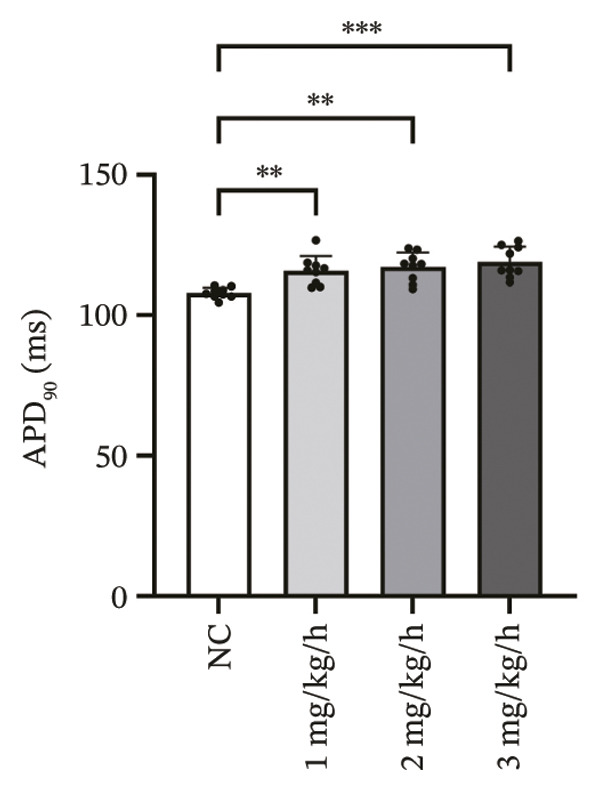
(f)

**Figure:**
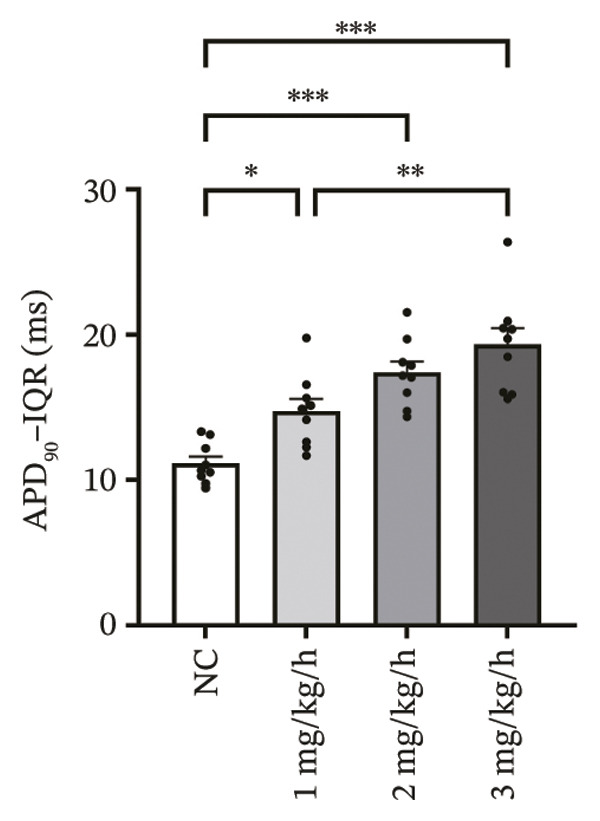
(g)

**Figure:**
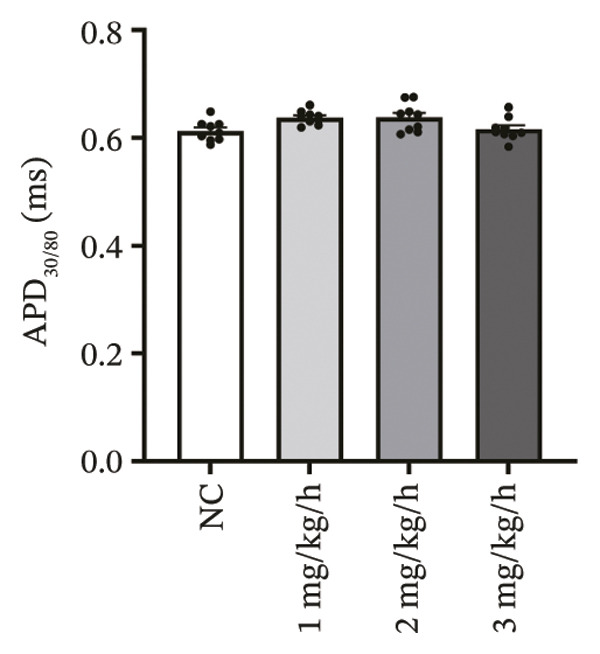
(h)

**Figure:**
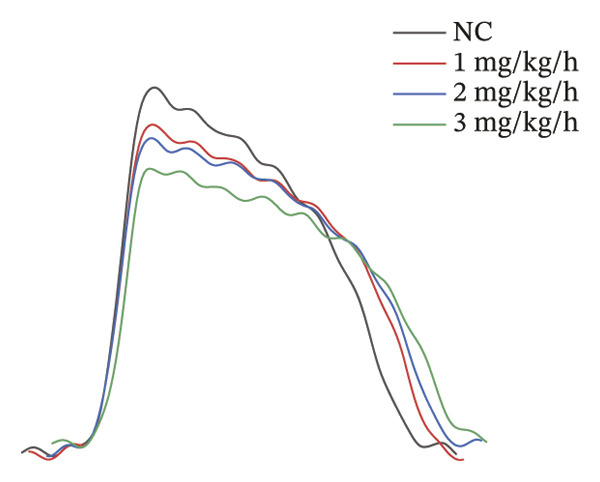
(i)

CaT analysis showed concordant changes. Active time was significantly prolonged in the 3 mg/kg/h group compared with 1 mg/kg/h (41.20 ± 2.07 vs 33.89 ± 1.35, *p* = 0.001). CaT rise time did not differ significantly among groups (NC: 29.11 ± 2.58 ms; 1 mg/kg/h: 28.32 ± 1.45 ms; 2 mg/kg/h: 35.91 ± 1.42 ms; 3 mg/kg/h: 32.28 ± 2.83 ms; *p* > 0.05), and the velocity likewise showed no significant difference (NC: 2.482 ± 0.060 mm/ms; 1 mg/kg/h: 2.744 ± 0.131 mm/ms; 2 mg/kg/h: 2.564 ± 0.085 mm/ms; 3 mg/kg/h: 2.502 ± 0.068 mm/ms; *p* > 0.05). CTD_90_ increased at remimazolam doses (NC: 116.20 ± 1.04 ms; 1 mg/kg/h: 121.93 ± 0.46 ms, *p* = 0.007; 2 mg/kg/h: 121.74 ± 1.15 ms, *p* = 0.003; 3 mg/kg/h: 125.63 ± 1.15 ms, *p* < 0.001). CTD_90_‐IQR was significantly higher at 3 mg/kg/h than at 1 mg/kg/h (10.33 ± 0.55 vs 8.14 ± 0.09, *p* = 0.005) (Figure 4). Together, these findings demonstrate dose‐related prolongation and increased spatial dispersion in both AP and CaT dynamics, consistent with an arrhythmogenic electrophysiological substrate at higher remimazolam exposure.

FIGURE 4Effects of remimazolam on calcium transient (CaT) characteristics in Langendorff‐perfused guinea pig hearts. All CaT signals were recorded under standardized electrical pacing at 4.5 Hz. (a–c) Representative maps of CaT activation time (a), calcium transient duration at 90% recovery (CTD_90_) (b), and CaT rise time (c) under NC conditions and during remimazolam perfusion at 1, 2, and 3 mg/kg/h. (d–h) Group comparisons of CaT activation time (d), CaT conduction velocity (e), CaT rise time (f), CTD_90_ (g), and CTD_90_ interquartile range (IQR) (h) across groups. (i) Overlaid representative CaT traces from each group illustrating dose‐dependent changes in calcium handling kinetics. NC (*n* = 9), 1 mg/kg/h (*n* = 6), 2 mg/kg/h (*n* = 9), or 3 mg/kg/h (*n* = 9). Statistical comparisons were performed using one‐way ANOVA followed by Tukey’s post hoc test. Data are presented as mean ± SEM; ^∗^ *p* < 0.05, ^∗∗^ *p* < 0.01, and ^∗∗∗^ *p* < 0.001.

**Figure:**
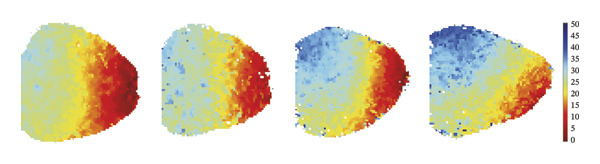
(a)

**Figure:**
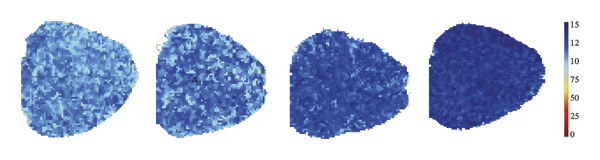
(b)

**Figure:**
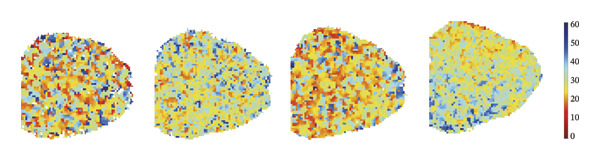
(c)

**Figure:**
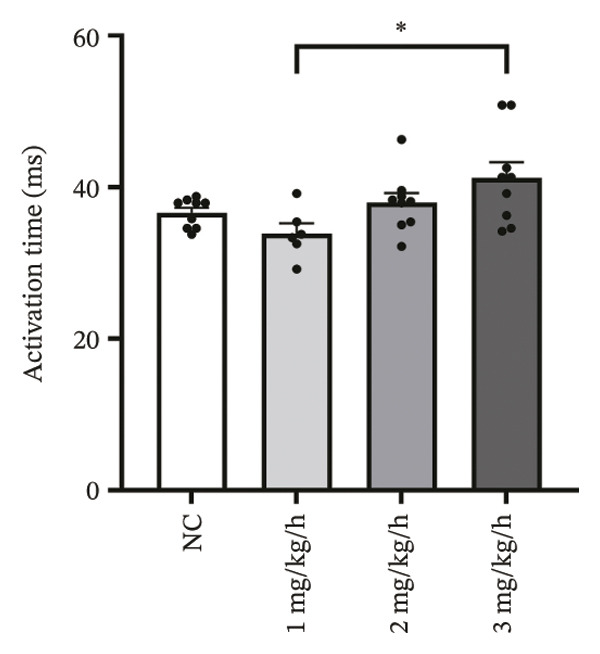
(d)

**Figure:**
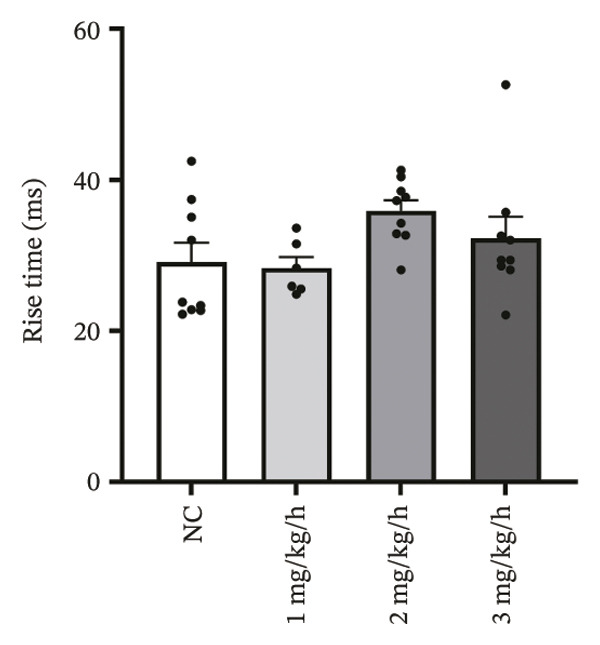
(e)

**Figure:**
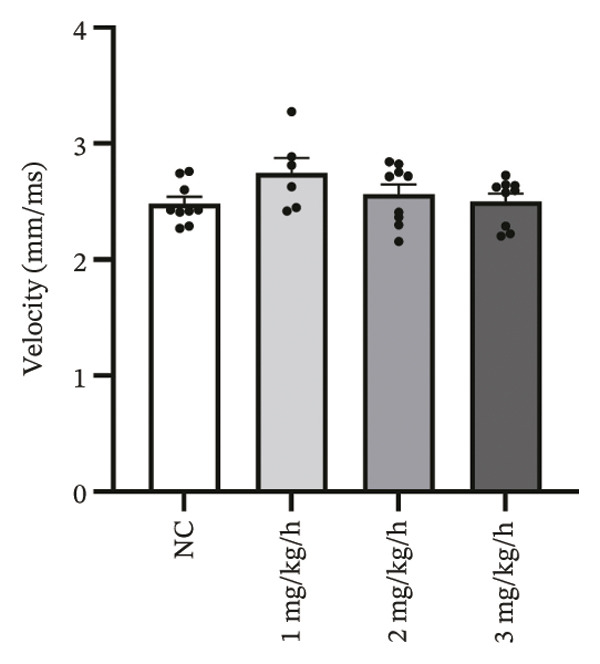
(f)

**Figure:**
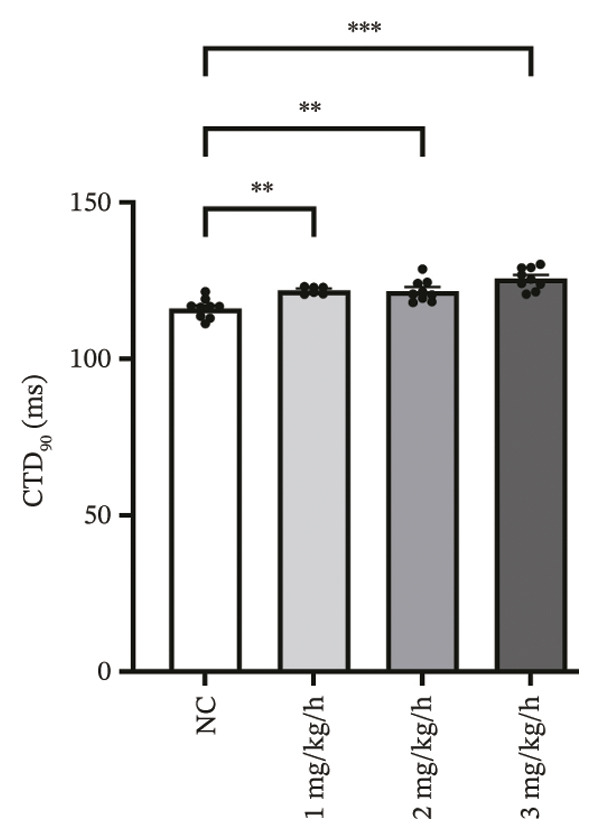
(g)

**Figure:**
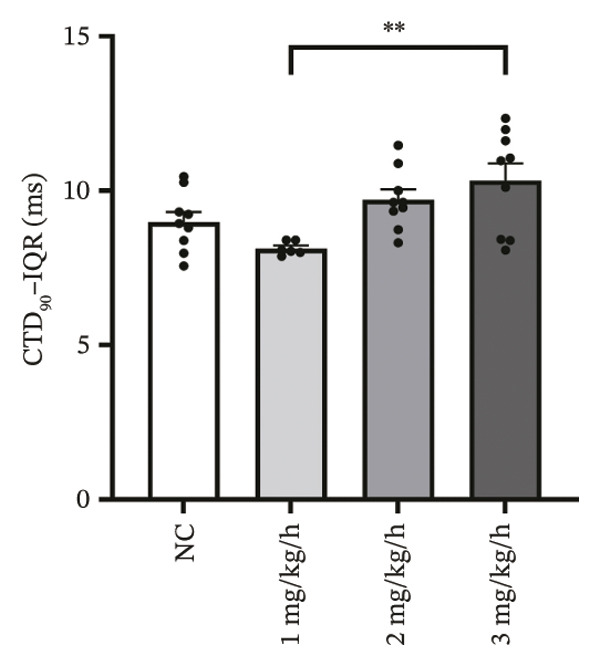
(h)

**Figure:**
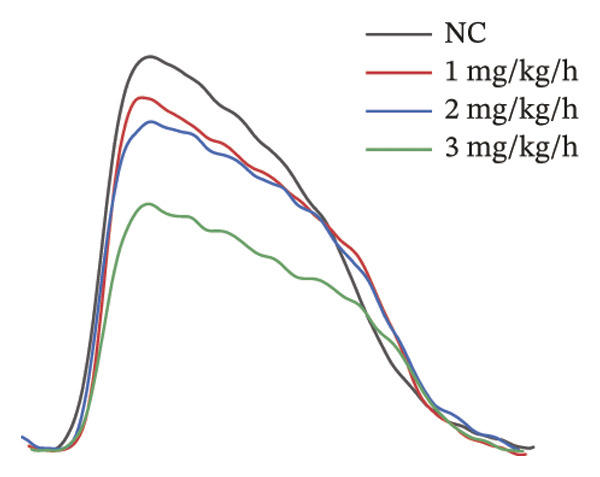
(i)

### 3.4. High‐Dose Remimazolam Delays Late Repolarization in hiPSC‐CMs

Using whole‐cell patch‐clamp recordings, the electrophysiological effects of remimazolam at plasma‐equivalent concentrations corresponding to 1, 2, and 3 mg/kg/h (500, 1000, and 1500 ng/mL) were evaluated in hiPSC‐CMs. Depolarization‐related properties were comparable across groups, with no significant differences in APA, Vmax, or MP (all *p* > 0.05). In contrast, late repolarization was delayed, with APD_90_ increasing from 116.72 ± 16.00 ms (NC) to 128.49 ± 16.90 ms (500 ng/mL), 173.46 ± 26.99 ms (1000 ng/mL), and 198.93 ± 23.97 ms (1500 ng/mL). Tukey’s post hoc test identified a significant prolongation only at 1500 ng/mL versus NC (mean difference: 82.22 ms, *p* = 0.04), whereas all other pairwise comparisons were not significant (all *p* > 0.05). Early and mid‐repolarization metrics remained statistically unchanged after correction: APD_10_ was 8.16 ± 3.00, 5.58 ± 2.38, 13.52 ± 3.84, and 11.56 ± 3.07 ms; APD_50_ was 58.17 ± 11.66, 58.49 ± 12.30, 90.27 ± 22.70, and 97.23 ± 24.79 ms; APD_10–50_ was 50.01 ± 10.57, 52.91 ± 11.53, 76.75 ± 20.75, and 85.66 ± 23.10 ms; and APD_50–90_ was 58.55 ± 7.49, 70.00 ± 8.89, 83.20 ± 22.60, and 101.70 ± 20.17 ms for NC, 500, 1000, and 1500 ng/mL, respectively (all *p* > 0.05) (Figure 5). Collectively, these data indicate that, within the clinically relevant range tested, remimazolam selectively prolongs late repolarization (APD_90_) in hiPSC‐CMs at the highest concentration, while depolarization and earlier repolarization indices remain unchanged.

FIGURE 5Effects of remimazolam on action potential characteristics in hiPSC‐CMs assessed by whole‐cell patch‐clamp. Remimazolam was applied at plasma‐equivalent concentrations corresponding to 1, 2, and 3 mg/kg/h (500, 1000, and 1500 ng/mL). (a–c) Group comparisons of action potential amplitude (APA) (a), maximum upstroke velocity (Vmax) (b), and resting membrane potential (MP) (c), showing no significant differences among groups. (d–g) Action potential durations at different repolarization levels, including APD_10_ (d), APD_50_ (e), APD_90_ (f), and the triangulation index (APD_50–90_) (g). (h) Quantification of APD_90_ interquartile range (IQR) across groups. (i) Representative overlaid action potential traces illustrating prolonged late repolarization at higher remimazolam concentrations. *n* = 10 (control), 9 (500 ng/mL), 8 (1000 ng/mL), and 8 (1500 ng/mL) cells. Statistical comparisons were performed using one‐way ANOVA followed by Tukey’s post hoc test (or Kruskal–Wallis with Dunn’s multiple comparisons, as appropriate). Data are presented as mean ± SEM; ^∗^ *p* < 0.05, ^∗∗^ *p* < 0.01, and ^∗∗∗^ *p* < 0.001.

**Figure:**
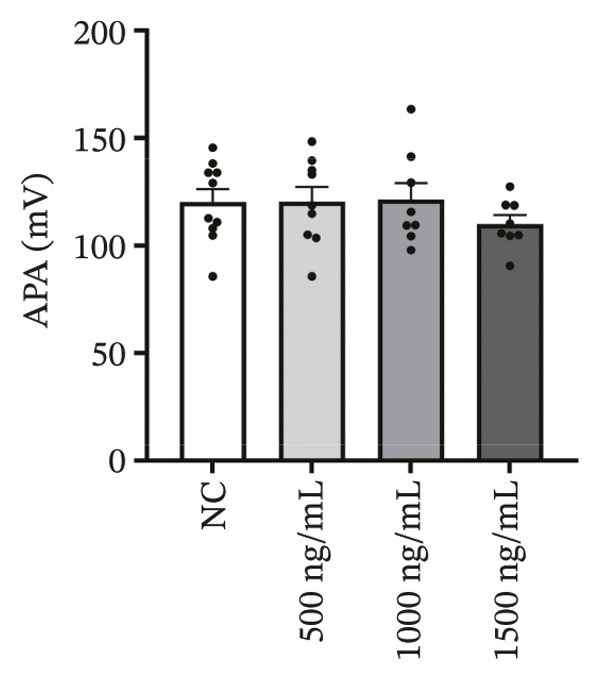
(a)

**Figure:**
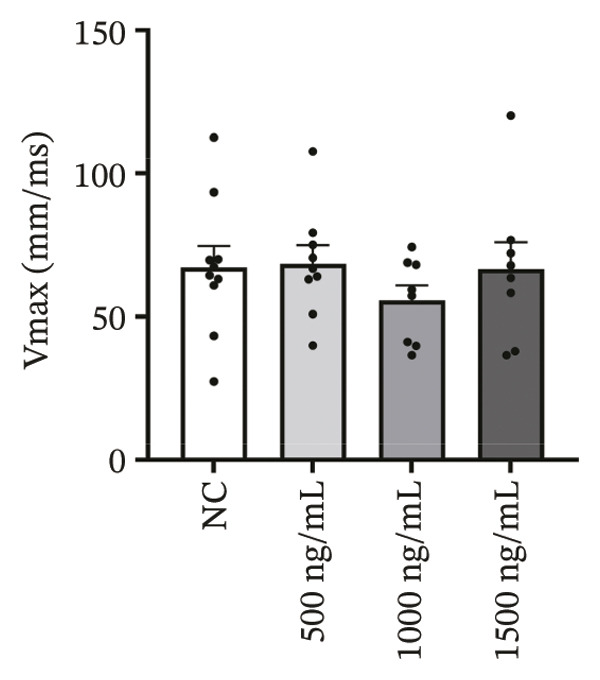
(b)

**Figure:**
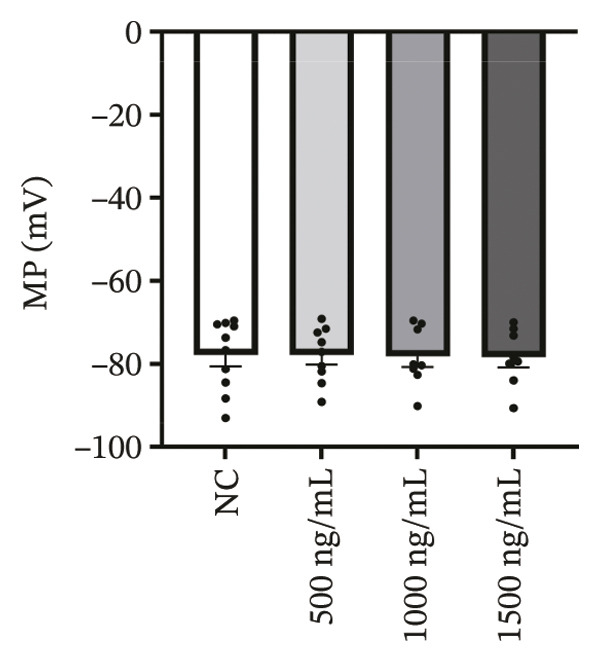
(c)

**Figure:**
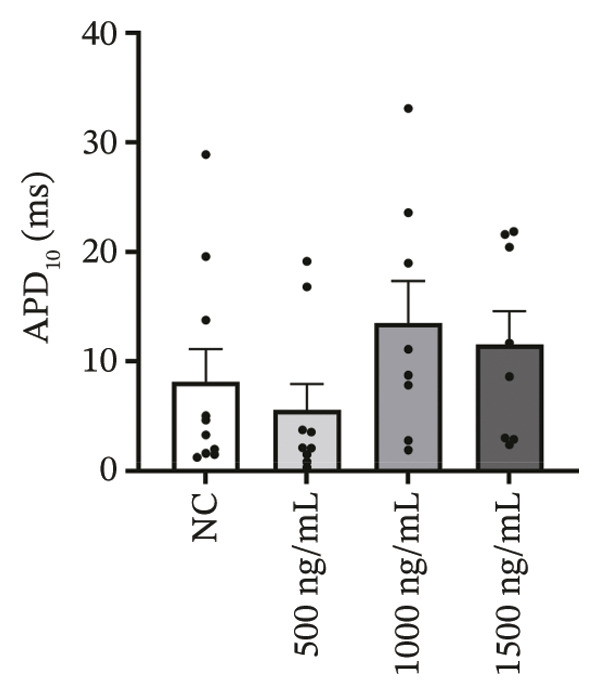
(d)

**Figure:**
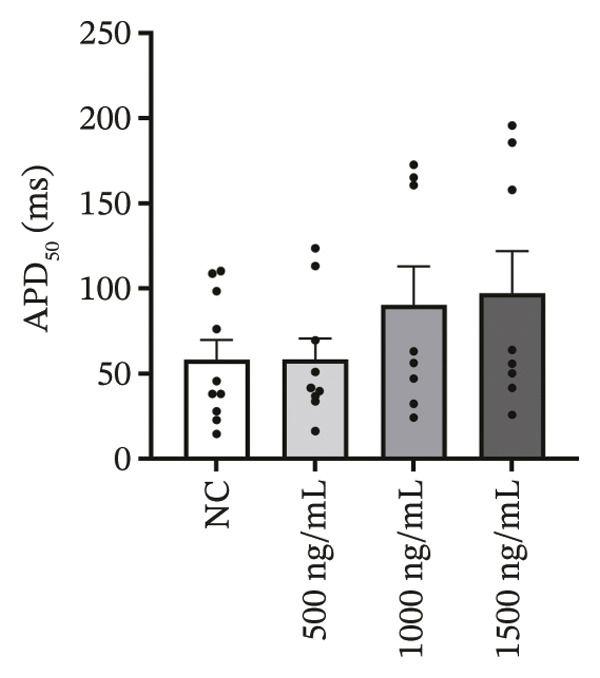
(e)

**Figure:**
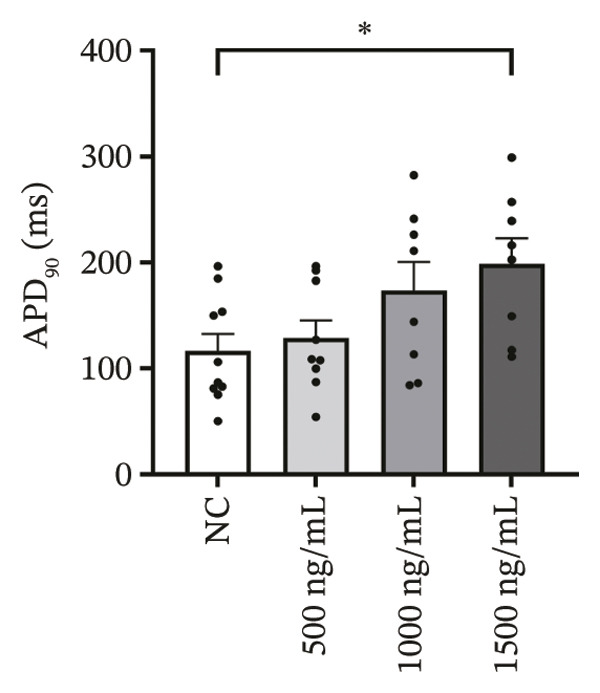
(f)

**Figure:**
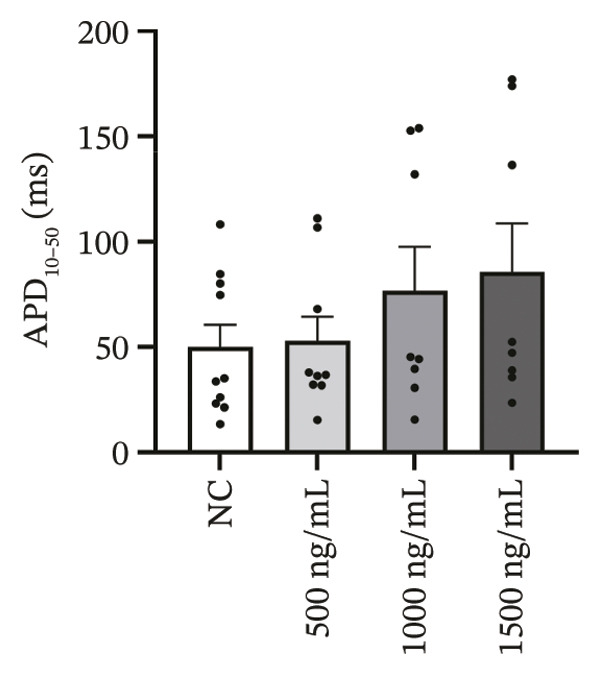
(g)

**Figure:**
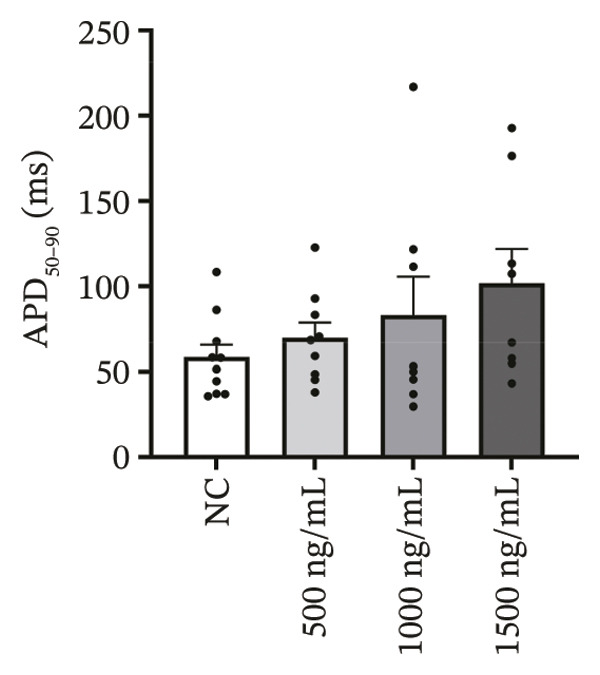
(h)

**Figure:**
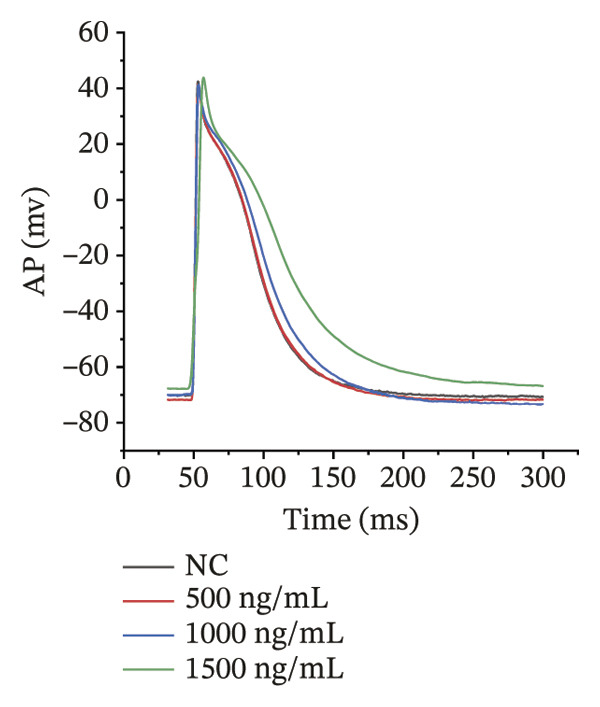
(i)

## 4. Discussion

To our knowledge, this study is among the first to evaluate the cardiac electrophysiological safety of remimazolam across multiple experimental levels, linking preclinical findings to potential clinical implications. We combined multichannel electrical mapping, optical mapping, and whole‐cell patch‐clamp recordings in hiPSC‐derived cardiomyocytes to assess remimazolam effects from the tissue level down to single cells. Our main findings are as follows. (1) Remimazolam prolonged the PR interval only at the highest dose and increased T wave duration in a dose‐dependent manner, consistent with delayed atrioventricular conduction and altered ventricular repolarization. (2) Higher doses increased activation time and spatial conduction heterogeneity, despite no significant change in average CV. (3) Optical mapping showed prolonged APD_90_ and CTD_90_, together with increased repolarization dispersion at 2–3 mg/kg/h. (4) In hiPSC‐CMs, APD_90_ was selectively prolonged at 1500 ng/mL, while depolarization‐related parameters remained unchanged.

We observed broadly consistent electrophysiological changes across multiple experimental models, including isolated Langendorff‐perfused guinea pig hearts and hiPSC‐CMs, which strengthen the overall reliability of our findings. On surface ECG, remimazolam at clinically relevant infusion rates (2‐3 mg/kg/h) significantly increased T wave duration, while PR prolongation was most apparent at the highest dose, suggesting potential delays in atrioventricular conduction and ventricular repolarization. At the same time, none of the three concentrations tested in our study produced significant changes in QT, QTc, or QT/QTc, which is consistent with prior studies in healthy volunteers reporting no QT prolongation and no clinically meaningful QTc effect with remimazolam [8, 18]. However, tissue‐level optical and electrical mapping revealed prolonged APD_90_ and CTD_90_ together with increased spatial dispersion of repolarization at these infusion rates. At the cellular level, 1500 ng/mL remimazolam, a concentration within the clinically achievable range, prolonged APD_90_ in hiPSC‐CMs without affecting depolarization‐related parameters (APD_10_, APD_50_, APA, and Vmax).

These observations suggest that the absence of QT/QTc changes on surface ECG does not necessarily exclude subtle electrophysiological effects at higher exposure, particularly those related to late repolarization dynamics and spatial heterogeneity. Clinically, such effects may be more relevant in patients with preexisting arrhythmias or increased repolarization vulnerability, where multiple stressors and concomitant factors can amplify otherwise modest changes.

Activation time in high‐density mapping is commonly defined at the point of maximal negative dV/dt and reflects the timing of local depolarization, whereas CV represents the average speed of impulse propagation across the mapped region [19]. In our study, higher remimazolam exposure prolonged activation time and increased indices of spatial nonuniformity, including dispersion, inhomogeneity index, and absolute inhomogeneity [20–22]. Notably, mean CV was reduced at the highest dose, indicating that the drug effect was not limited to localized delays but could also slow overall propagation under higher exposure. At the same time, the marked rise in heterogeneity metrics suggests that impaired conduction was not spatially uniform. Prior mapping work shows that conduction delay often emerges as direction‐ and region‐dependent slowing, with greater discrepancies between longitudinal and transverse propagation, which can increase spatial gradients even when average values alone do not fully capture the substrate [19, 23, 24]. Mechanistic studies have also described activation–repolarization interactions, where changes in local activation may reshape repolarization dynamics and amplify spatial dispersion [25]. Together, these features provide a plausible link between the mapping findings and the T‐wave broadening and heterogeneity observed on ECG, reflecting an underlying conduction substrate that becomes more vulnerable at higher remimazolam exposure [26].

The preferential prolongation of late repolarization (APD_90_ and CTD_90_) observed in this study likely reflects combined effects on repolarizing currents and intracellular Ca^2+^ handling. First, previous in vitro studies have demonstrated that remimazolam can inhibit the rapid delayed rectifier potassium current (I_Kr_), mediated by the hERG channel, which plays a crucial role in Phase 3 repolarization of cardiomyocytes [18, 27, 28]. Reduced I_Kr_ would be expected to slow late repolarization and thereby prolong APD, which may increase susceptibility to repolarization abnormalities such as early afterdepolarizations (EADs) under permissive conditions [29]. Second, the dose‐dependent prolongation of CTD_90_ and increase in CTD_90_‐IQR suggest that disrupted intracellular Ca^2+^ handling may contribute to delayed repolarization [30]. CTD_90_ reflects cytosolic calcium decay, regulated primarily by reuptake into the sarcoplasmic reticulum via sarcoplasmic/endoplasmic reticulum Ca^2+^‐ATPase (SERCA) and extrusion through the Na^+^/Ca^2+^ exchanger (NCX) [30–32]. Impairments in either pathway can prolong intracellular Ca^2+^ clearance and sustain membrane depolarization during late repolarization [33]. Elevated CTD_90_‐IQR further indicates regional disparities in calcium handling, which may underlie the spatial heterogeneity in repolarization [34]. Interestingly, Urabe et al. reported that remimazolam induced intracellular Ca^2+^ elevation via endoplasmic reticulum release in noncardiomyocyte models under supratherapeutic conditions [35]. While their experimental context differs from ours, this finding supports the possibility that remimazolam may affect intracellular calcium dynamics. Taken together, these mechanisms provide a plausible explanation for the late‐repolarization prolongation and the increased electrical heterogeneity observed at higher remimazolam exposure in our tissue‐level recordings.

This study has several limitations. First, we did not directly measure plasma concentrations of remimazolam, limiting precise correlation to clinical dosing. Second, although we observed electrophysiological effects consistent with previous reports on hERG inhibition and calcium handling alterations, we did not perform direct ionic current recordings [18, 35]. Thus, while altered repolarizing potassium currents (e.g., I_Kr_, I_Ks_, and I_K1_), prolonged L‐type calcium current (I_Ca-L_), or enhanced late sodium current (I_Na-Late_) may contribute to the observed phenotypes, direct evidence of remimazolam’s specific effects on these channels is lacking. Future studies employing targeted patch‐clamp or pharmacological blockade techniques are warranted to elucidate the precise ionic mechanisms involved.

In conclusion, this study systematically evaluated the electrophysiological effects of remimazolam across multiple experimental levels, including ECG, optical mapping in isolated guinea pig hearts, and whole‐cell patch‐clamp recordings in hiPSC‐derived cardiomyocytes. We found that remimazolam, at higher clinically relevant concentrations, selectively prolonged late repolarization (APD_90_ and CTD_90_), increased conduction dispersion and repolarization heterogeneity, and induced measurable changes in ECG parameters, including T wave duration and PR interval at the highest dose. These findings were consistent across cellular and tissue levels and suggest that remimazolam may alter the spatiotemporal dynamics of cardiac excitation, potentially creating a proarrhythmic substrate under specific conditions. Although no significant QT and QTc prolongation was observed, our data suggest that remimazolam produces only limited electrophysiological perturbations within the clinically relevant range tested; therefore, routine use is unlikely to materially increase arrhythmic risk. Clinicians may still individualize dosing and ECG surveillance in patients with preexisting conduction or repolarization abnormalities, electrolyte imbalance, or concomitant QT‐risk medications.

## Author Contributions

Zijun Wang is the first author. Ying Cao contributed equally and is recognized as a co‐first author. Hong Gao is the designated corresponding author.

Zijun Wang, Ying Cao, and Hong Gao conceptualized and designed the research protocol. Zijun Wang, Gao Su, and Yanyan Feng performed the animal experiments. Zijun Wang and Ying Cao drafted the manuscript. Rongfeng Yang and Xue Bai verified the authenticity of the raw data and performed statistical analyses. Hong Gao supervised the ethical approval process and ensured compliance with institutional animal care standards. Zijun Wang acquired funding for the study. Hong Gao and Ying Cao also critically reviewed the manuscript and oversaw data interpretation. All authors contributed to manuscript revision.

## Funding

This study was supported by the Science and Technology Program of Guiyang Health Commission (Grant [2020] Zhukejian Technology Contract No. 003) and the Guizhou Administration of Traditional Chinese Medicine (Grant No. QZYY‐2021‐134).

## Disclosure

All authors reviewed the manuscript and approved its submission. The funders had no role in study design, data collection and analysis, decision to publish, or preparation of the manuscript.

## Ethics Statement

All experimental procedures were approved by the Animal Ethics Committee of Third Affiliated Hospital of Guizhou Medical University, China (No. 2021A010), and were conducted in accordance with national guidelines and the NIH Guide for the Care and Use of Laboratory Animals (8th edition).

## Conflicts of Interest

The authors declare no conflicts of interest.

## Data Availability

The data generated in the present study may be requested from the corresponding author.

## References

[1] Zaballos M. , Del Blanco B. , Sevilla R. et al., Differential Effects of Sevoflurane and Propofol on Swine Cardiac Conduction System, Veterinary Anaesthesia and Analgesia. (2019) 46, no. 3, 344–351, 10.1016/j.vaa.2018.11.007, 2-s2.0-85062231699. 30833141

[2] Wutzler A. , Huemer M. , Boldt L.-H. et al., Effects of Deep Sedation on Cardiac Electrophysiology in Patients Undergoing Radiofrequency Ablation of Supraventricular Tachycardia: Impact of Propofol and Ketamine, Europace: European Pacing, Arrhythmias, and Cardiac Electrophysiology. (2013) 15, no. 7, 1019–1024, 10.1093/europace/eut025, 2-s2.0-84879557572. 23407634

[3] Staikou C. , Stamelos M. , and Stavroulakis E. , Impact of Anaesthetic Drugs and Adjuvants on ECG Markers of Torsadogenicity, British Journal of Anaesthesia. (2014) 112, no. 2, 217–230, 10.1093/bja/aet412, 2-s2.0-84892696254. 24305646

[4] Khaleghi M. , Sarchahi A. A. , and Mehrjerdi H. K. , Effects of Ketamine, Propofol and Isoflurane on Electrocardiographic Variables in Clinically Healthy Dogs Premedicated with Medetomidine and Midazolam, Veterinary Research Forum: An International Quarterly Journal. (2024) 15, 187–194. 38770200 10.30466/vrf.2024.2008055.3954 PMC11102798

[5] Yildiz M. , Yilmaz Ak H. , Oksen D. , and Oral S. , Anesthetic Management in Electrophysiology Laboratory: A Multidisciplinary Review, Journal of Atrial Fibrillation. (2018) 10, no. 5, 10.4022/jafib.1775. PMC6006978 29988243

[6] Pesic M. , Schippers F. , Saunders R. , Webster L. , Donsbach M. , and Stoehr T. , Pharmacokinetics and Pharmacodynamics of Intranasal Remimazolam-A Randomized Controlled Clinical Trial, European Journal of Clinical Pharmacology. (2020) 76, no. 11, 1505–1516, 10.1007/s00228-020-02984-z. 32886178 PMC7557484

[7] Doi M. , Hirata N. , Suzuki T. , Morisaki H. , Morimatsu H. , and Sakamoto A. , Safety and Efficacy of Remimazolam in Induction and Maintenance of General Anesthesia in High-Risk Surgical Patients (ASA Class III): Results of a Multicenter, Randomized, Double-Blind, Parallel-Group Comparative Trial, Journal of Anesthesia. (2020) 34, no. 4, 491–501, 10.1007/s00540-020-02776-w. 32303884

[8] Eisenried A. , Schüttler J. , Lerch M. , Ihmsen H. , and Jeleazcov C. , Pharmacokinetics and Pharmacodynamics of Remimazolam (CNS 7056) After Continuous Infusion in Healthy Male Volunteers: Part II. Pharmacodynamics of Electroencephalogram Effects, Anesthesiology. (2020) 132, no. 4, 652–666, 10.1097/aln.0000000000003102. 31972657

[9] Worthington M. T. , Antonik L. J. , Goldwater D. R. et al., A Phase Ib, Dose-Finding Study of Multiple Doses of Remimazolam (CNS 7056) in Volunteers Undergoing Colonoscopy, Anesthesia & Analgesia. (2013) 117, no. 5, 1093–1100, 10.1213/ane.0b013e3182a705ae, 2-s2.0-84887070779. 24108261

[10] Borkett K. M. , Riff D. S. , Schwartz H. I. et al., A Phase IIa, Randomized, Double-Blind Study of Remimazolam (CNS 7056) Versus Midazolam for Sedation in Upper Gastrointestinal Endoscopy, Anesthesia & Analgesia. (2015) 120, no. 4, 771–780, 10.1213/ane.0000000000000548, 2-s2.0-84930922239. 25502841

[11] Rex D. K. , Bhandari R. , Desta T. et al., A Phase III Study Evaluating the Efficacy and Safety of Remimazolam (CNS 7056) Compared With Placebo and Midazolam in Patients Undergoing Colonoscopy, Gastrointestinal Endoscopy. (2018) 88, no. 3, 427.e6–437.e6, 10.1016/j.gie.2018.04.2351, 2-s2.0-85049754079. 29723512

[12] Wesolowski A. M. , Zaccagnino M. P. , Malapero R. J. , Kaye A. D. , and Urman R. D. , Remimazolam: Pharmacologic Considerations and Clinical Role in Anesthesiology, Pharmacotherapy: The Journal of Human Pharmacology and Drug Therapy. (2016) 36, no. 9, 1021–1027, 10.1002/phar.1806, 2-s2.0-84990184988. 27496519

[13] Stöhr T. , Colin P. J. , Ossig J. et al., Pharmacokinetic Properties of Remimazolam in Subjects With Hepatic or Renal Impairment, British Journal of Anaesthesia. (2021) 127, no. 3, 415–423, 10.1016/j.bja.2021.05.027. 34246461

[14] Lee S. , Seo J. , Kim D. Y. et al., Comparison of Hemodynamic Parameters Based on the Administration of Remimazolam or Sevoflurane in Patients Under General Anesthesia in the Beach Chair Position: A Single-Blinded Randomized Controlled Trial, Journal of Clinical Medicine. (2024) 13, no. 8, 10.3390/jcm13082364. PMC11051199 38673637

[15] Sekiguchi R. , Kinoshita M. , Kawanishi R. et al., Comparison of Hemodynamics During Induction of General Anesthesia With Remimazolam and Target-Controlled Propofol in Middle-Aged and Elderly Patients: A Single-Center, Randomized, Controlled Trial, BMC Anesthesiology. (2023) 23, no. 1, 10.1186/s12871-023-01974-9. PMC9830695 36624371

[16] Oh E. J. , Chung Y. J. , Lee J.-H. et al., Comparison of Propofol vs. Remimazolam on Emergence Profiles After General Anesthesia: A Randomized Clinical Trial, Journal of Clinical Anesthesia. (2023) 90, 10.1016/j.jclinane.2023.111223. 37506483

[17] Camm A. J. , Clinical Trial Design to Evaluate the Effects of Drugs on Cardiac Repolarization: Current State of the Art, Heart Rhythm. (2005) 2, no. 11, S23–S29, 10.1016/j.hrthm.2004.09.019, 2-s2.0-27744587040. 16253928

[18] Kleiman R. B. , Darpo B. , Thorn M. , Stoehr T. , and Schippers F. , Potential Strategy for Assessing QT/QTc Interval for Drugs that Produce Rapid Changes in Heart Rate: Electrocardiographic Assessment of the Effects of Intravenous Remimazolam on Cardiac Repolarization, British Journal of Clinical Pharmacology. (2020) 86, no. 8, 1600–1609, 10.1111/bcp.14270. 32144789 PMC7373701

[19] Kanai A. and Salama G. , Optical Mapping Reveals that Repolarization Spreads Anisotropically and is Guided by Fiber Orientation in Guinea Pig Hearts, Circulation Research. (1995) 77, no. 4, 784–802, 10.1161/01.res.77.4.784, 2-s2.0-0029101792. 7554126

[20] Dong X. , Tse G. , Hao G. , and Du Y. , Heterogeneities in Ventricular Conduction Following Treatment With Heptanol: A Multi-Electrode Array Study in Langendorff-Perfused Mouse Hearts, Life. (2022) 12, no. 7, 10.3390/life12070996. PMC9321110 35888085

[21] Ma Y. , Cao Y. , Gao H. et al., Sevoflurane Improves Ventricular Conduction by Exosomes Derived from Rat Cardiac Fibroblasts After Hypothermic Global Ischemia-Reperfusion Injury, Drug Design, Development and Therapy. (2023) 12, no. 17, 1719–1732, 10.2147/DDDT.S408595. PMC10275581 37333963

[22] Lammers W. J. , Schalij M. J. , Kirchhof C. J. , and Allessie M. A. , Quantification of Spatial Inhomogeneity in Conduction and Initiation of Reentrant Atrial Arrhythmias, American Journal of Physiology. (1990) 259, no. 4, H1254–H1263, 10.1152/ajpheart.1990.259.4.h1254. 1699438

[23] Kelly A. , Ghouri I. A. , Kemi O. J. et al., Subepicardial Action Potential Characteristics are a Function of Depth and Activation Sequence in Isolated Rabbit Hearts, Circulation: Arrhythmia and Electrophysiology. (2013) 6, no. 4, 809–917, 10.1161/CIRCEP.113.000334, 2-s2.0-84884520291. 23733913

[24] Krul S. P. J. , Berger W. R. , Smit N. W. et al., Atrial Fibrosis and Conduction Slowing in the Left Atrial Appendage of Patients Undergoing Thoracoscopic Surgical Pulmonary Vein Isolation for Atrial Fibrillation, Circulation: Arrhythmia and Electrophysiology. (2015) 8, no. 2, 288–295, 10.1161/circep.114.001752, 2-s2.0-84932155852. 25673630

[25] Hanson B. , Sutton P. , and Elameri N. , Interaction of Activation-Repolarization Coupling and Restitution Properties in Humans, Circulation: Arrhythmia and Electrophysiology. (2009) 2, no. 2, 162–170, 10.1161/CIRCEP.108.785352, 2-s2.0-70349451834. 19808461

[26] Arteyeva N. V. , Dispersion of Ventricular Repolarization: Temporal and Spatial, World Journal of Cardiology. (2020) 12, no. 9, 437–449, 10.4330/wjc.v12.i9.437. 33014291 PMC7509993

[27] Ponte M. L. , Keller G. A. , and Di Girolamo G. , Mechanisms of Drug Induced QT Interval Prolongation, Current Drug Safety. (2010) 5, no. 1, 44–53, 10.2174/157488610789869247, 2-s2.0-77950855126. 20210718

[28] Al Sayed Z. R. , Pereira C. , Le Borgne R. et al., CAVIN1-Mediated hERG Dynamics: A Novel Mechanism Underlying the Interindividual Variability in Drug-Induced Long QT, Circulation. (2024) 150, no. 7, 563–576, 10.1161/circulationaha.123.063917. 38682330

[29] Maruyama M. , Lin S.-F. , Xie Y. et al., Genesis of Phase 3 Early Afterdepolarizations and Triggered Activity in Acquired long-QT Syndrome, Circulation: Arrhythmia and Electrophysiology. (2011) 4, no. 1, 103–111, 10.1161/circep.110.959064, 2-s2.0-79953791303. 21078812 PMC3045276

[30] Sun H. , Song J. , Li K. et al., Increased β1-Adrenergic Receptor Antibody Confers a Vulnerable Substrate for Atrial Fibrillation via Mediating Ca2+ Mishandling and Atrial Fibrosis in Active Immunization Rabbit Models, Clinical Science. (2023) 137, no. 2, 195–217, 10.1042/CS20220654. 36597894 PMC9885845

[31] Hwang J. , Kim T. Y. , Terentyev D. et al., Late INa Blocker GS967 Supresses Polymorphic Ventricular Tachycardia in a Transgenic Rabbit Model of Long QT Type 2-PubMed, Circulation: Arrhythmia and Electrophysiology. (2020) 13, no. 8, 10.1161/CIRCEP.118.006875. PMC10626560 32628505

[32] Wang L. , Morotti S. , Tapa S. et al., Different Paths, Same Destination: Divergent Action Potential Responses Produce Conserved Cardiac Fight-or-Flight Response in Mouse and Rabbit Hearts, Journal of Physiology. (2019) 597, no. 15, 3867–3883, 10.1113/JP278016, 2-s2.0-85068758991. 31215643 PMC6675632

[33] Eisner D. A. , Caldwell J. L. , Kistamás K. , and Trafford A. W. , Calcium and Excitation-Contraction Coupling in the Heart, Circulation Research. (2017) 121, no. 2, 181–195, 10.1161/circresaha.117.310230, 2-s2.0-85021898173. 28684623 PMC5497788

[34] Tomek J. , Hao G. , Tomková M. et al., β-Adrenergic Receptor Stimulation and Alternans in the Border Zone of a Healed Infarct: An Ex Vivo Study and Computational Investigation of Arrhythmogenesis, Frontiers in Physiology. (2019) 10, 10.3389/fphys.2019.00350, 2-s2.0-85066441471. PMC6450465 30984029

[35] Urabe T. , Miyoshi H. , Narasaki S. et al., Characterization of Intracellular Calcium Mobilization Induced by Remimazolam, A Newly Approved Intravenous Anesthetic, PLoS One. (2022) 17, no. 2, 10.1371/journal.pone.0263395. PMC8806057 35104283

